# Physiological and Emotional Responses of Disabled Children to Therapeutic Clowns: A Pilot Study

**DOI:** 10.1093/ecam/neq008

**Published:** 2011-03-13

**Authors:** Shauna Kingsnorth, Stefanie Blain, Patricia McKeever

**Affiliations:** ^1^Bloorview Research Institute, Bloorview Kids Rehab, University of Toronto, Toronto, Ontario, Canada; ^2^Lawrence S. Bloomberg Faculty of Nursing, University of Toronto, Toronto, Ontario, Canada; ^3^Department of Paediatrics, Faculty of Medicine, University of Toronto, Toronto, Ontario, Canada; ^4^Institute of Biomaterials and Biomedical Engineering, University of Toronto, Toronto, Ontario, Canada; ^5^SickKids Research Institute, The Hospital for Sick Children, Toronto, Ontario, Canada

## Abstract

This pilot study examined the effects of Therapeutic Clowning on inpatients in a pediatric rehabilitation hospital. Ten disabled children with varied physical and verbal expressive abilities participated in all or portions of the data collection protocol. Employing a mixed-method, single-subject ABAB study design, measures of physiological arousal, emotion and behavior were obtained from eight children under two conditions—television exposure and therapeutic clown interventions. Four peripheral autonomic nervous system (ANS) signals were recorded as measures of physiological arousal; these signals were analyzed with respect to measures of emotion (verbal self reports of mood) and behavior (facial expressions and vocalizations). Semistructured interviews were completed with verbally expressive children (*n* = 7) and nurses of participating children (*n* = 13). Significant differences among children were found in response to the clown intervention relative to television exposure. Physiologically, changes in ANS signals occurred either more frequently or in different patterns. Emotionally, children's (self) and nurses' (observed) reports of mood were elevated positively. Behaviorally, children exhibited more positive and fewer negative facial expressions and vocalizations of emotion during the clown intervention. Content and themes extracted from the interviews corroborated these findings. The results suggest that this popular psychosocial intervention has a direct and positive impact on hospitalized children. This pilot study contributes to the current understanding of the importance of alternative approaches in promoting well-being within healthcare settings.

## 1. Introduction

Since therapeutic clowning began in North America in 1986, it has become a popular practice in acute and rehabilitation hospitals worldwide [1–5] and increasingly is thought to play an important complementary role in healthcare [2, 6, 7]. In contrast to the more familiar circus clown whose goal is to entertain, Therapeutic Clowns aim to promote patients' well-being by supporting their expressions of control and emotion using pleasurable and playful techniques. By creating contexts that enable individualized, improvisational, often humorous social exchanges, Therapeutic Clowns alter the social and physical hospital milieu to enhance the physical and mental well-being of patients and care providers [2, 6–10].

Studies of therapeutic clowning have shown that this intervention facilitates verbal and nonverbal communication [7, 10]; improves mood and attitude [6, 11, 12]; increases expressions of emotion such as laughter, joy and humor [7, 13–16]; supports empowerment and active role-reversal [13] and is perceived as a valuable complementary therapy by patients, families and care providers [12, 15, 16]. While these results are encouraging, they must be interpreted with caution due to the limitations of their predominantly qualitative or evaluative study designs. Studies using experimental designs with children have focused exclusively on the effectiveness of goal-directed clowning to distract from pain and stress before or during invasive medical procedures. The results of these studies are mixed and understanding limited to acute settings [17–20]. Hence, understanding of how therapeutic clowning affects the physical and mental well-being of hospitalized chronically ill and/or disabled children and their care providers is limited.

Children in long-term rehabilitation settings experience far more than invasive medical procedures; prolonged restricted activities, disempowerment, lack of personal control and lengthy separations from their families contribute greatly to their stress and anxiety [21, 22]. Although the effectiveness of therapeutic clowning in enabling children to cope in this context is unknown, this intervention is likely to have significant positive effects. This hypothesis is supported by studies that have shown that therapeutic clowning enhances emotional and behavioral responses [6, 7, 11–16]. Positive changes in emotional responding arising from humor and laughter have been correlated with increased pain thresholds and immunity, inversely correlated with stress hormone levels and linked to positive health [23–29].

Gathering data to validate these effects in long-term care settings, however, is complicated by the fact that many children have a variety of conditions that prevent them from responding in ways that are typical of nondisabled children. These conditions include severe cerebral palsy, traumatic brain injury or brainstem stroke and render many unable to move, gesture or speak. Hence, measures that rely on speech, typical movements or invasive techniques like blood sampling are neither feasible nor ethically justifiable [30–32]. As a result of these challenges, such children are often excluded from research participation. However, recent research indicates that autonomic nervous system (ANS) state or physiological signal trends can be successfully correlated with emotional states and thus can provide an alternative and noninvasive response measure [33–37]. Given the importance of inclusivity to our understanding of best therapeutic practice, the goal of this pilot study is to investigate physiological signals as a measure of responsiveness to arts-based interventions, specifically, therapeutic clowning.

Emotions modulate many processes controlled by the ANS including posture, skin temperature and moisture, respiration and muscle tension [33–37]. When anxious, hands become cold and clammy, hearts race and breathing becomes shallow and rapid. These changes arise from changes in electrodermal activity (EDA), skin temperature, respiration and blood volume pulse (BVP). More specifically, EDA is a measure of the electrical conductance of the skin. Changes in EDA occur as a result of cholinergenic stimulation of the sweat glands, causing them to release ion-rich sweat, thus increasing the overall conductivity [38, 39]. Fingertip skin temperature is influenced by the cutaneous microcirculation of the hand. Among the fingertip vascular structures are ateriovenous anatomoses, which are innervated by sympathetic nerve fibers that react to stimulation of the ANS [40]. The respiratory and cardiovascular systems are dually innervated by both branches of the ANS; stimulation from the sympathetic branch causes increased functioning in both systems and stimulation from the parasympathetic branch reverses this behavior [35]. These changes can be spontaneously, reflexively or voluntarily generated by a variety of internal or external arousal stimuli. The interested reader is referred to [35] for a more detailed description of physiological responding.

In 1983, Eckman demonstrated that five measures of the ANS (heart rate, left and right hand temperatures, EDA and forearm muscle tension) could distinguish between six emotional states: surprise, disgust, sadness, anger, fear and happiness [36]. More recently, in the field of affective computing, computers have been trained to classify ANS signals to distinguish among a variety of emotions. For example, Picard et al. [34] used facial muscle tension, heart rate, skin conductance and respiration to distinguish between self-reported emotions (neutral, anger, hatred, grief, platonic love, romantic love, joy and reverence) to an accuracy of 81%. Similarly, Katsis et al. [41] used the same set of signals to distinguish between high stress, low stress, disappointment, euphoria and neutral states to an accuracy of 86%. These findings suggest that ANS signals are reliable indices of specific emotions [33–37, 41–43].

However, while the results of these studies are promising, the physiological signals were collected from nondisabled adults in a controlled setting, where a constrained number of emotions were induced and validated through self-report and behavioral observation [34, 36, 37, 41–43]. The ability for physiological signals to detect unconstrained, mixed sets of emotions in naturalistic environments is still under debate. This problem is further confounded when these signals are gathered from disabled children, whose physiological systems may respond with different patterns than nondisabled adults. Thus, to attempt to understand the effects of therapeutic clowning on disabled children experiencing prolonged hospitalization, we augmented their physiological signals with direct observations of their behaviors, self-reported mood and third-party observations of interactions. Based on current understanding, we hypothesize that children would demonstrate positive, differential physiological, behavioral and emotional responses to Therapeutic Clowns.

## 2. Methods

### 2.1. Setting and Design

This study was conducted in a large urban pediatric hospital that provides long-term rehabilitative and complex continuing care to children with a range of physical, cognitive and/or developmental congenital or acquired disabilities. Many of the children have conditions that affect the somatic branch of their ANS, causing decreased or involuntary muscular control, which in some cases renders them unable to move or to speak. For these children, many physical and physiological modalities by which emotion can be expressed are differently affected or rendered uninformative. Given the potential for idiosyncratic patterns of responding, between and/or within-subject group designs were not appropriate—hence a single-subject research design was adopted [44]. A mixed methods approach was used to capture the children's physiological, behavioral and emotional responses to therapeutic clowning.

### 2.2. Participants

Fourteen inpatients between the ages of 4 and 21 years were recruited to participate in this study. Ethical approval was received from the relevant institution, and written consent was obtained from children and/or their guardians. Children were excluded if they had a visible breach of the epidermis or dermis on their arms or their hands. According to these criteria, and as a result of equipment malfunction (1), drop-out (1), experimenter error (1) and collection difficulties related to small hand size (2) and/or spasticity (1), objective quantitative data from eight inpatients (4 male, mean age = 10.7 years, SD = 5.0) were retained. Five of these children were verbally expressive and had the cognitive skills needed to contribute self-report and interview data. An additional two verbally expressive children from the total sample also participated in the interview yielding a sub-set of seven participants (4 male, mean age = 10.7 years, SD = 2.9) for this component. Thus, the final sample comprised 10 children with primary diagnoses that included Arterio-venous Malformation, Guillan–Barré Syndrome, Macrophage Activation Syndrome, Transverse Myelitis, Cerebral Palsy and Encephalopathy. While one child had been hospitalized for over 5 years, the average length of hospitalization for the remaining nine children was 52 days (SD = 37). Six of the children had been exposed to therapeutic clowning before participating in this study.

### 2.3. Intervention

As part of an established professional Therapeutic Clown program, Dr Flap and Ricky visit three inpatient units of a 75 bed facility two afternoons a week in a duo-clown partnership. Dr Flap has performed professionally as clown and actor for 17 years in theatre and circus realms; Ricky has performed professionally as an actor for over 10 years. They have also performed for 3 and 5 years, respectively, as therapeutic clown practitioners in large urban pediatric hospitals. Costumed in only a red nose and equipped with a multitude of tools (including music and rhythm, movement and physical comedy, storytelling and role-reversal, and games), the clowns endeavor to empower children during short individualized visits (e.g., 10–15 min).

A children's television program was selected as a familiar control stimulus that provided similar audiovisual stimulation (e.g., loud, colorful, musical, humorous, etc.), permitted individual choice and that would be accessible to all children irrespective of cognitive abilities or developmental status.

### 2.4. Data Collection

Data were collected over 4 days of alternating interventions (ABAB) (Table 1). On control days (A), children watched a television show of their choice, and on intervention days (B), children received routine therapeutic clowning interventions. The study took place in each child's room at a consistent time to control for circadian rhythms in ANS responses. Before the commencement of this study, the clowns toured the units with mock physiological recording equipment to familiarize children. The research assistant further demonstrated the equipment and responded to concerns on the first day of data collection. For nonverbal children, a parent or a nurse familiar to the child assisted with set-up. No signs of distress or fear were noted among any of the participants.

During data collection, children were either comfortably seated or lying down and instructed to minimize their movements. Each session consisted of 20 min of data collection beginning with a 5 min baseline rest period (0 min ≤ *t* ≤ 5 min), 10 min of the scheduled intervention (5 min ≤ *t* ≤ 15 min) and a 5 min postintervention rest-period (15 min ≤ *t* ≤ 20 min). The baseline rest period ensured that children had acclimatized to the physiological recording equipment before the introduction of the intervention. The length of the intervention window was predetermined by standard clowning practice and is consistent with similar studies examining change in physiological responding among children [45–48].

### 2.5. Measures

In recognition of the children's diagnostic complexity and their diversity in verbal and physical expressive vocabularies, data collection tools were selected that enabled the inclusion of all inpatient children.

#### 2.5.1. Physiological Responses

**Figure 1:**
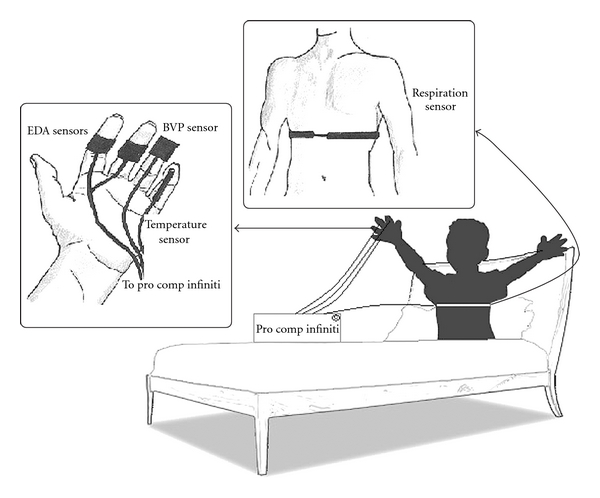
Illustration of the experimental setup for physiological signal data collection.

As outlined earlier, EDA, skin temperature, respiration and BVP are strongly associated with emotive responding [41–43]. These four physiological signals were collected with noninvasive sensors and the ProComp Infiniti, a data acquisition system from Thought Technology. To monitor EDA, two gel-less Ag/Ag-Cl sensors were attached to the medial phalange of the index and middle finger of the child's nondominant hand. To monitor BVP, an infrared sensor was loosely secured with velcro to his/her fourth finger and to monitor skin temperature, a surface thermistor was attached to his/her fifth finger with breathable tape. Finally, a piezoelectric belt was comfortably positioned around the child's thorax to monitor respiratory signals. The positioning of these sensors is illustrated in Figure 1. All sensors were sampled at a frequency of 256 Hz. Continuous-time recording was used during each 20 min collection session to control for temporal variations and transient changes in the signals and to determine each child's pattern of physiological responding [49].

#### 2.5.2. Behavioral Responses

Six facial and vocal expressions that are commonly associated with emotions of a strong positive or negative valence were tracked by one of two trained observers for the duration of data collection. The observers had previous experience with disabled children; with data collection methods (physiological and observational); and completed a joint training session to ensure reliability in coding. Positive valence expressions included laughing and smiling while negative valence expressions included yelling (without smiling), sighing, crying and grimacing [50, 51]. Each minute of the data collection period was divided into 20 s responses for a total of 60 intervals. The observer recorded the presence of behaviors during each 20 s interval.

#### 2.5.3. Emotional Responses

Feeling Faces Cards were used to facilitate verbal children's self-report of mood [52]. These cards present 20 exaggerated expressions of different valences on ethnically diverse male and female children's faces. Each day, children were asked at *t* = 0, 5, 15 and 20 min to select the card(s) in response to the question “How do you feel right now?”

#### 2.5.4. Verbal Responses

At the end of the 4 days of data collection, verbal children participated in a brief semistructured interview. They responded to the following five questions:

1. How do you feel on the days when Dr Flap and Ricky visit you?
2. How do you feel on the days when the clowns do not visit?
3. What things do you like about the clowns?
4. What things do you not like about the clowns?
5. What would you tell your friend/sibling about Dr Flap and Ricky?

#### 2.5.5. Nurses' Observations of Children's Responses

Thirteen nurses participated in short semistructured interviews to explore their perceptions and experiences of therapeutic clowning and observations of clown-child interactions. They responded to the following question: “Do you think the children feel and/or behave differently when the clowns visit?”

## 3. Results

### 3.1. Physiological Responses

The four physiological signals were analyzed using a custom-made multivariate change detection algorithm designed to detect patterns of physiological signal change (S Blain, S Kingsnorth, T Chau. Unpublished algorithm 2010. Toronto, ON: Bloorview Research Institute). For each physiological signal, a representative feature was extracted for every second of data collected. Features extracted during the initial 5 min rest period (Period 1) were used to describe the multivariate mean, range and variability of the pattern of physiological responding unique to that child while he/she was at rest. Features extracted during the subsequent 10 min intervention period (Period 2) were compared against those generated during the resting state (Period 1). Time points in which the extracted features did not belong to the class of the child's resting patterns were counted and characterized, such that the analysis of each intervention yielded two outcome measures: (i) the total number of outliers (T), in other words, the number of seconds where the child's physiological signals did not fall within resting patterns and (ii) the average contribution of each physiological signal to the outliers generated during that intervention (*C*_avg_).

Both outcome measures were compared between television and clown interventions using regression analyses (logistic and linear, respectively, for *T* and *C*_avg_). To address intrasubject variability, a stringent criterion was adopted; children were classified as “frequency” responders if *T* was significantly different between the two television control days and the two clown intervention days (*P* < .05). Similarly, children were classified as “pattern” responders if any of their physiological signals demonstrated a significantly different pattern of response (i.e., if *C*_avg_ was significantly different) between the two television intervention days and the two clown intervention control days (*P* < .05). Classification was not exclusive; children could be classified as both a frequency and pattern responder.

Findings from the regression analyses are presented in Table 2. It is evident that no two children demonstrated the same pattern or combination of responsive physiological signals. All eight children experienced one or more significant physiological change in response to therapeutic clowning in comparison to watching television. The six classified as “frequency” responders all demonstrated significantly higher frequencies of arousal during Therapeutic Clown exposure relative to television exposure. Among the six children classified as “pattern” responders, there was variability in the dominating physiological signals and in the direction of change. For two, exposure to therapeutic clowning decreased their baseline levels of responding. For example, Participant 5 experienced a drop in fingertip temperature and a slowing of breathing rate. In contrast, Participants 1, 2, 6 and 7 all experienced increases in skin temperature either independently or in combination with another physiological signal.

### 3.2. Behavioral Responses

Behavioral responses were obtained from seven of the eight children, and a range of facial and vocal expressions were displayed. For each child, each behavioral response was normalized by taking the difference between the total frequency for Period 1 (see Table 1; *n* = 15 intervals) and the average total frequency for Period 2 (*n* = 30 trials/2) for each session. Logistic regression analysis was used to determine whether behavioral differences were significantly different (*P* < .05) between the television and clowning interventions.

As outlined in Table 3, seven of the eight children demonstrated positive and negative behavioral expressions of emotion. Using a moderately stringent criterion, cells are highlighted for all children who displayed one or more pairs of significant differences in expressive behavior between sessions (*P* < .05). Significant increases in smiling and laughing and decreases in grimacing were found. No differences between interventions were found for negative vocal expressions (i.e., yelling, crying and sighing); in general, negative expressions of emotion were low in frequency.

### 3.3. Emotional Responses

To explore changes in emotion, each child's mood report was assigned a positive or negative valence. The most common responses across the four data collection sessions were reports of being happy (*n* = 22), content (*n* = 8), excited (*n* = 3) or surprised (*n* = 3). Negative mood reports included being tired (*n* = 12), bored (*n* = 8) or hungry (*n* = 3); no expressions of fear were reported. These emotions were reported individually or in combination (e.g., “happy and content” or “tired and bored”). Changes in overall valence and strength before (*t* = 5) and after (*t* = 15) Period 2 were determined for each day.  Table 4 displays the results of a visual inspection of these changes. Participants 3, 5 and 8 did not have a reliable means of communication and were unable to self-report, therefore are excluded from this table. Despite 4 missing cells on clown days among the verbal children, the overall trend demonstrated is a positive change in mood following exposure to the clowns and no change in mood following exposure to a children's television show.

### 3.4. Self- and Nurse Observations of Responses

The interview data were transcribed verbatim and analyzed using qualitative data analysis software [53]. Data were explored using a range of techniques including content analysis and testing of emergent themes. A coding scheme was developed based on the study's goals and questions as well as themes emerging from the interviews. Transcripts were coded for descriptive content, such as nurses' descriptions of children's behavior and children's description of therapeutic clown behavior. Coded texts were analyzed for patterns and themes that illuminated the children's responses to therapeutic clowning.

#### 3.4.1. Clown-Child Observations and Interviews

The children's interviews yielded very positive descriptions of their responses to therapeutic clowning; six of the seven verbal children said they feel “happy” and five said they feel “excited” when the clowns visit. On days when the clowns do not visit, three children said they feel “sad”, one is “bored”, two “happy” and one child feels “ok”. It is noteworthy that when children expressed negative emotions, they did so within the frame of reference of the clown's game; for example, two children described not liking specific clown behaviors: “Ricky is naughty and flooded the bathroom” [9]; “… they don't listen … they are bad. Sometimes Ricky misbehaves and I give him a timeout on the bed” [6]. These reports indicate that the children are enthusiastically engaged with the clowns while simultaneously disapproving the clown's naughty behavior.

#### 3.4.2. Nurses' Observations of Children's Responses

The nurses' descriptions of children's responses are also overwhelmingly positive. Almost all the words used by the nurses to describe the children's moods when interacting with the clowns suggest increased energy, happiness and excitement; and some nurses perceive the children to be more relaxed when the clowns are present. Some behavior that nurses ascribed to the children were quite complex, such as deliberately manipulating the clowns or pretending to feel an emotion as part of the play. Referring to a nonverbal child, one nurse said “Even if she doesn't laugh or smile when they are right there singing, when they go to another child she will smile when they leave. Sometimes she pulls that on them too and they think she doesn't want them, she will then smile, but she doesn't want them to see that”. When examined in the context of verbal children's self reports about disciplining the clowns, and the clowns' intent to give children power, this description may reflect children's multilayered emotional experiences—such as enjoyment in expressing anger or pleasure in acting bored.

## 4. Discussion

### 4.1. Impact of Therapeutic Clowns on Children in a Rehabilitation Setting

Despite their popularity, alternative and complementary health interventions such as therapeutic clowning are at risk of being cut in times of fiscal restraint. In the absence of empirical evidence supporting their benefits to physical and mental well-being, they are considered nonessential and difficult to justify. This study begins to address this need by determining the physiological, behavioral and emotional effects of therapeutic clowning on children in a rehabilitation hospital.

Across all children, significant differences in patterns of ANS responses were found in response to therapeutic clowning as compared to exposure to a children's television program. Although physiological measures were the only measure which could be ubiquitously obtained from all participants, the interpretation of these signals is entirely dependent on complementary measures of response. While previous work has demonstrated that physiological signals are emotion specific, the heterogeneity of this population yields individual-specific patterns of physiological change. This is clearly illustrated in comparing the results presented in Tables 2 and 3. While Participants 1, 2, 4, 5 and 6 all demonstrated greater positive behavioral changes in response to therapeutic clowning than to television, no two children demonstrated the same patterns of physiological change. We can ascribe positive and negative valence to physiological responses that are accompanied by behavioral and/or verbal responses, but in the absence of this complementary information, we cannot accurately interpret these results. Despite the intersubject variability in ANS response patterns, the direct and third-party observations of increased smiling and laughing, decreased grimacing and positive changes in mood strongly suggest that the physiological changes observed can be considered to reflect positive responses.

These findings complement and expand current understanding of this popular art-based intervention to include disabled children who are experiencing prolonged and/or repeated hospitalizations [6–20]. Furthermore, they demonstrate the value of using a broad spectrum of techniques and sources, especially for heterogeneous populations commonly encountered in rehabilitation hospitals. Only five participants were able to contribute all three types of data (i.e. physiological, behavioral and verbal) as operationalized in the current study. The need to use a multipronged approach to facilitate the inclusion of profoundly disabled children may underlie the paucity of empirical research exploring their experiences of the world.

### 4.2. Interrelation among Response Modalities

In addition to enhancing understanding of the effects of therapeutic clowning, our multipronged approach further highlights the interrelationships among expressive modalities and the strength of triangulation. For example, nurses described an emotion to be present (such as happiness or anticipation), and when pressed to elaborate, described behavioral indicators such as flapping arms, concentrating more intently or smiling. Thus, nurses perceived very clear connections between these children's emotional and behavioral responses; one nurse even connected a child's emotional state to physiological signals and specifically referred to changes in respiration and blood oxygen saturation as indicators of the child's pleasure in response to therapeutic clowning. These qualitative findings are consistent with understanding of emotion as a modulator of almost all forms of verbal and human communication [34]. More importantly, they showcase the limits of conventional definitions of expressive behaviors.

As a result of the broad spectrum of impairments in our sample, the range of observed behaviors exceeded the six behaviors selected to represent overt expressions of emotion. For example, a significant response may be a muscle twitch in one child and a rapid and wild wheelchair ride in another child. Certainly, typical behaviors, such as laughing, eye contact and moving towards or following the clowns, were described by many nurses, but other described behaviors that included flapping arms and legs, rolling around in bed, longer durations of directed eye-gaze or making sounds. Further complicating interpretation of emotional expressions were reports suggesting that multiple emotions were experienced simultaneously, such as pleasure in feeling angry in the context of disciplining the clowns. By using narrow indices of happiness and unhappiness, the full range of emotion-specific behaviors evidenced may not have been captured. Further work involving disabled and/or chronically ill children should move away from facial and vocal expressions of emotion typical of nondisabled children and instead use responses that reflect the particular emotional expressions of individual children [30].

### 4.3. Children with Severe and/or Multiple Disabilities

More broadly, findings from this study have implications for understanding how profoundly disabled children experience the world. Participants 3, 5 and 8 do not have a reliable physical or verbal means of expressing themselves and decoding their responses to stimuli is very difficult. The reader can get a clear understanding of how daunting this task is by reviewing the limits of the conventional measures of affect we employed. These children were not able to verbally report, either by interview or by choosing a Feeling Faces card, their mood or their perception of the clowns. Participant 8, in particular, either did not engage in normative expressions of emotion or his/her behavior during the therapeutic clowning interventions was not significantly different from typical patterns. Although nurses expressed confidence in being able to “read” the responses of these children—several answered “definitely” and “you can certainly tell” in response to questions about whether a movement or behavior signified a given mood or desire—data from the conventional measures alone does not support their interpretations. This “knowledge” is likely a function of the nurses' extensive training in caring for such children and familiarity with their complex needs.

In the absence of overt and consistent displays of emotion or preference, it is easy for those not intimately familiar with these children's idiosyncratic nuanced expressions to assume that they are nonresponsive. Table 2, however, presents an alternate story—all three of these children were “frequency” responders—with significant increases in physiological responding to the Therapeutic Clowns that were not merely a function of audiovisual stimulation. Two children were also “pattern” responders displaying differential patterns of responding. Thus, in spite of a lack of evidence from conventional measures, the physiological data clearly demonstrated that therapeutic clowning had an effect on these children.

While the valence of these reactions remains unverifiable in the absence of overt behaviors or self-report, these results illustrate the value of using physiological signals as a means of garnering insight into the emotional state of children with profound and/or severe impairments. Furthermore, they enhance our understanding of the different ways people respond to their environments and broaden current definitions of participation and inclusion. For example, changes in physiological responding may be a means of determining preferences for meaningful and enjoyable activities among children with profound disabilities and ensuring varied social interactions, engagement and positive stimulation—factors that contribute significantly to positive emotions and quality of life [54].

### 4.4. Strengths and Limitations

Although small sample sizes and heterogeneous populations are typically considered study limitations, we consider these circumstances as strengths. By using a single-subject research design, this pilot project was elevated from a case study to a rigorous experimental design. Normalization of the data relative to baseline for each data collection session further ensured that differences in responses were the result of the intervention and not a consequence of day-to-day changes in health status (e.g. pain, medication and lack of sleep) among the heterogeneous population. Multiple data sources and measures allowed for a triangulation of findings—a process of particular relevance for individuals with limited expressive modalities. Finally, continuous ANS recording avoided potential pitfalls associated with single time point recordings. While some information can be gleaned from the latter data collection approach, caution must be exercised in its interpretation, particularly with heterogeneous populations. EDA, for example, demonstrates temporal variation throughout the day and is subject to the circadian rhythms of the body [49]. Furthermore, the salient information in EDA patterns can only be gleaned through continuous recording.

Although small in numbers, the current findings strongly suggest that the changes in physiological responding were not a function of audiovisual stimulation, expressive ability or of a pre-existing relationship with the clowns—as significant differences were seen for all children. The possibility remains however that these changes are the result of a live interaction with another person. Identification of a suitable control for a therapeutic clown challenged the authors; in the end, selection of a children's television show provided the greatest amount of control and consistency across participants. It should be noted that despite attempts to keep the presence of the observers neutral and unobtrusive, many of the children had a difficult time ignoring their presence and engaged them in conversation. Furthermore, the third-party observations provided by the nurses suggest that the presence of therapeutic clowns on the unit changes the children's behavior and mood in ways that are not typically seen in other interactions between children and caregivers.

Future work should build on these strengths by replicating the current findings with a larger sample size to permit a more detailed exploration of physiological responding among disabled children under different environmental conditions. The inclusion of broader definitions and more sophisticated assessments of “nontraditional” behavioral indices of emotion are strongly recommended to minimize observer bias and facilitate validation and interpretation of the ANS signals.

## 5. Conclusions

In the recent years, there has been a rapid expansion of therapeutic clowning programs in healthcare settings. Despite the popularity of this intervention, there has been little systematic investigation of its effects. The current study is valuable in its efforts to address gaps in the literature. First and foremost, it is the first study to explore and demonstrate that therapeutic clowning has a direct physiological impact on children. Second, it expands the range of healthcare settings investigated in the literature to include rehabilitation and focuses on children with chronic conditions experiencing prolonged hospitalizations. Third, the behavioral, self-report and third-party observational data support existing evidence that therapeutic clowning has an overall positive effect on mood and well-being—even on profoundly disabled children. These findings offer a promising platform to support the continued investigation of therapeutic outcomes among heterogeneous populations.

## Funding

Funding for this project was provided by a Bloorview Research Institute Seed Grant (08-054).

## Figures and Tables

**Table 1:** Overview of study design.

Intervention (duration)	Period 1: Baseline (5 min)	Period 2: Intervention (10 min)	Period 3: Rest (5 min)
A	Rest	Television (TV)	Rest
B	Rest	Therapeutic Clowning (TC)	Rest

**Table 2:** Results of the regression analyses of the ANS data.

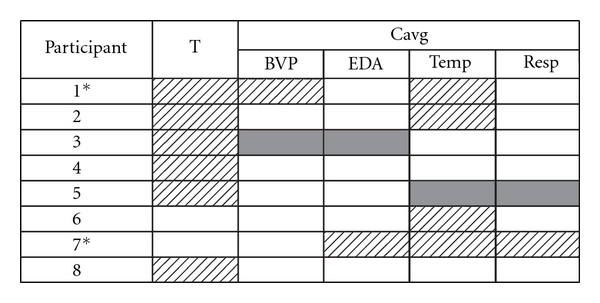

Highlighted cells represent significant differences between AA and BB conditions (*P* < .05). Solid cells indicate a reduction in physiological responding while hatched cells represent an increase relative to baseline levels. *Denotes partial data set (i.e., AAB).

**Table 3:** Results of the logistic regression analysis of behavioral responses.

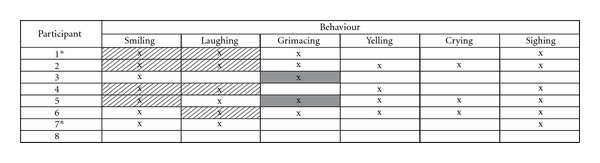

All cells marked by “x” denote observed behaviors. Highlighted cells represent significant effects (*P* < .05); solid cells indicate a reduction in frequency of behavioral responding while hatched cells represent an increase relative to baseline levels. *Denotes partial data set (i.e., AAB).

**Table 4:** Results of a visual inspection of change in emotional response pre to post intervention.

Participants	Clown intervention		Television intervention	
	Day 1	Day 2	Day 1	Day 2
1		↑	**=**	**=**
2		**=**	**=**	**↓**
4	**↑**	**↑**	**=**	**=**
6	**↑**		**↑**	**↑**
7	**↑**		**=**	**=**

Blank cells denote missing data. The direction of the arrow in the cell represents the direction of change in mood pre to post intervention: ↑ represents positive change, **=** represents no change, **↓** represents negative change.
